# Early postoperative neurocognitive complications in elderly patients: comparing those with and without preexisting mild cognitive impairment– a prospective study

**DOI:** 10.1186/s12877-024-04663-5

**Published:** 2024-01-22

**Authors:** Pawit Somnuke, Pensiri Srishewachart, Chalita Jiraphorncharas, Asamaporn Khempetch, Jirapa Weeranithan, Patumporn Suraarunsumrit, Varalak Srinonprasert, Arunotai Siriussawakul

**Affiliations:** 1 Department of Anesthesiology, Faculty of Medicine Siriraj Hospital, Mahidol University, 10700 Bangkok, Thailand; 2 Faculty of Medicine, Integrated Perioperative Geriatric Excellent Research Center, Siriraj Hospital, Mahidol University, 10700 Bangkok, Thailand; 3 Queen Sirikit National Institute of Child Health, 10400 Bangkok, Thailand; 4 Nakhon Pathom Hospital, 73000 Nakhon Pathom, Thailand; 5 Division of Geriatric Medicine, Department of Medicine, Faculty of Medicine Siriraj Hospital, Mahidol University, 10700 Bangkok, Thailand

**Keywords:** Diagnostic and Statistical Manual of Mental Disorders, Fifth Edition (DSM-5), Elderly, Mild cognitive impairment (MCI), Montreal Cognitive Assessment (MoCA), Postoperative cognitive dysfunction (POCD), Postoperative delirium (POD)

## Abstract

**Background:**

As societies age, increasing numbers of older adults undergo surgeries with anesthesia. Postoperative delirium (POD) and postoperative cognitive dysfunction (POCD) frequently occur in older surgical patients. Most of these patients already have preoperative mild cognitive impairment (MCI). However, the correlation between MCI and POD remains unclear. This study aimed to determine the incidence of POD in elderly patients with and without preexisting MCI.

**Methods:**

A prospective study enrolled patients aged 60 years and above scheduled for major surgeries between December 2017 and April 2022. Preoperative MCI was determined by a Montreal Cognitive Assessment (MoCA) score between 18 and 24. POD was diagnosed using criteria from the fifth edition of the Diagnostic and Statistical Manual of Mental Disorders (DSM-5). POCD was characterized by a MoCA score reduction of 2 or more points from the preoperative score. The primary outcome was the incidence of POD within the first 72 h postoperatively. Secondary outcomes encompassed other postoperative complications, including POCD.

**Results:**

The study comprised 223 elderly patients with MCI and 56 without MCI. The incidence of POD was 16.6% in the MCI group and 14.3% in the non-MCI group (*P* = 0.839). POCD occurred in 24.3% of MCI patients and 50% of non-MCI patients (*P* = 0.001). There were no significant differences in other postoperative complications between the groups. Postoperatively, the MCI group notably declined in visuospatial, attention, and orientation domains, while the non-MCI group declined in all domains except delayed recall.

**Conclusions:**

The incidence of POD was similar in the MCI and non-MCI groups. However, the non-MCI group demonstrated a higher incidence of POCD than the MCI group. This was identified by a reduction in postoperative MoCA scores for the visuospatial, naming, attention, language, abstraction, and orientation domains. These findings underscore the importance of postoperative cognitive assessments for both elderly patients with preexisting MCI and those with previously intact cognitive functions.

**Trial registration:**

This trial was retrospectively registered in the Thai Clinical Trials Registry on 15/01/2019 (registration number: TCTR20190115001).

**Supplementary Information:**

The online version contains supplementary material available at 10.1186/s12877-024-04663-5.

## Introduction

With socioeconomic development and medical advancements, the global population is rapidly increasing. The number of individuals aged 60 and older has increased exponentially, reaching 670 million in 2022 and accounting for 14% of the world’s population. This proportion is projected to climb to 26% by 2050. Approximately 60% of all individuals aged 60 years and above reside in Asia and the Pacific region [1]. Thailand stands out as one of the Asian nations with the most accelerated aging rate. Of Thailand’s 67 million inhabitants, 12 million (17.9%) are considered elderly. This proportion is expected to surge to 28% within the next decade [2].

As longevity may correlate with a rise in morbidities over time, conditions requiring medical or surgical intervention are major concerns for elderly individuals. It has been documented that 53% of all surgical procedures are performed in this population segment [3]. Frailty, a state of accumulated physiological deterioration across various organ systems in elderly individuals, has been reported to be a better predictor of perioperative adverse events than age alone [4–6]. Frailty has been linked to an increased risk of memory function deficits, including mild cognitive impairment (MCI) and dementia [7–10]. In the broader community, MCI is frequently diagnosed among elderly individuals, with an incidence rate of approximately 17% [11]. However, this rate can soar to 87% among nursing home residents [12]. Considering the high occurrence of MCI in the elderly, it is intriguing to explore its possible connection with adverse postoperative results.

Postoperative delirium (POD) is a life-threatening condition recognized by the American Geriatric Society as a prevalent postoperative complication [13]. Previous studies reported a POD incidence ranging from 10 to 20% for elective noncardiac surgeries and 15–50% for cardiac surgeries, starkly contrasting with the 2.5–3% delirium rate in the general population [14–18]. The characteristics of POD comprise the following attributes: (1) the sudden onset and fluctuation of symptoms, (2) inattention, (3) disorganized thinking, and (4) an altered level of consciousness. Delirium manifests in three distinct types: hyperactive, hypoactive, and mixed presentations. The highest incidence of postoperative delirium is observed within the initial three days following surgery [19]. The first-line therapy for the treatment of POD is a non-pharmacological approach, emphasizing the identification and management of underlying causes, and the elimination of precipitating factors for POD. This involves measures such as avoiding patient restraint, frequent patient reorientation, adjusting the surroundings, and promoting sleep. In case the etiology is unclear or non-pharmacologic interventions prove ineffective, antipsychotic medications—haloperidol, olanzapine, risperidone, and quetiapine—are recommended [20–22]. It remains uncertain whether patients with baseline MCI have a higher predisposition to develop POD. Prior studies have employed various tools to detect preexisting or preoperative MCI, such as the Montreal Cognitive Assessment (MoCA) test [23], Mini-Mental State Examination (MMSE) [24, 25], and Saint Louis University Mental Status Examination [26]. However, their findings lacked consensus on the correlation between MCI and a heightened POD incidence [27].

Postoperative cognitive dysfunction (POCD) is another prevalent postoperative complication requiring thorough pre- and postoperative assessments. Preoperative cognitive impairment has been shown to increase the incidence of POCD [28, 29]. However, the connection between preoperative MCI and POCD remains ambiguous due to limited studies and controversial findings [30–33].

This study primarily aimed to ascertain the incidence of POD in elderly patients with and without preexisting MCI. The secondary outcomes were the incidence of POCD and other postoperative complications.

## Methods

### Study design

This prospective, descriptive study was conducted at a university-affiliated hospital, Bangkok, Thailand. The research protocol received authorization from the Institutional Review Board of the Faculty of Medicine Siriraj Hospital, Mahidol University (approval number Si 515/2017). Data collection spanned from December 2017 to April 2022.

### Study population

We enrolled patients aged ≥ 60 who demonstrated comprehension of verbal and written Thai and were scheduled for major surgery under anesthesia. The surgeries encompassed by our study included neurological, cardiovascular-thoracic (CVT), colorectal, hepatobiliary, gynecological, urological, and orthopedic procedures.

Patients who declined participation or were unsuitable for neurocognitive assessment were excluded. Unsuitability for neurocognitive assessment was determined by an inability to comprehend Thai, severe visual or auditory impairments, psychological disorders that could interfere with the assessment process, or the occurrence of preoperative delirium. Prior to surgery and data collection, participants were given a comprehensive explanation of the study, and written informed consent was obtained.

### Data collection

This study was conducted under the POCD cohort project, which was approved by the Institutional Review Board (IRB) of the Faculty of Medicine Siriraj Hospital, Mahidol University (IRB approval number 016037003). Study data, including: (1) patients’ preoperative demographic information (sex, age, and educational attainment), comorbidities, preoperative neurocognitive status, benzodiazepine premedication, and the site and type of surgical procedure; (2) intraoperative data comprising the anesthesia technique used, benzodiazepine use, and specifics such as the episodes of hypotension, cardiac arrhythmias, blood transfusions, and the surgery’s duration; and (3) data on postoperative neurocognitive assessments and any complications for subsequent analysis were prospectively collected from electronic medical records by research assistants and medical experts. The data were recorded using Research Electronic Data Capture tools (REDCap) [34, 35], which is hosted at the Department of Anesthesiology, Faculty of Medicine Siriraj Hospital. All data were anonymously labeled with research identification code. The qualified research team, comprising doctors, nurses, psychologists, and research assistants, was assigned different data entry tasks according to their expertise. The quality and accuracy of the data were determined before further analysis by SPSS Statistics, version 26 (IBM Corp, Armonk, NY, USA).

### Assessment tools

The participants underwent pre- and postoperative evaluations using the following cognitive assessment tools. The assessors were blinded to the patients’ baseline cognitive statuses and were unaware of the cognitive test results from others.

#### Montreal cognitive assessment (MoCA)

The MoCA test comprises several domains of a neurocognitive battery, including: 1) Visuospatial/Executive: (a) Trail Making Test (1 point), (b) Copy Cube (1 point), and (c) Clock Drawing (3 points), 2) Naming (3 points), 3) Memory (no points), 4) Attention: (a) Digit Span (2 points), (b) Vigilance (1 point), and (c) Serial 7 Subtraction (3 points), 5) Language: (a) Sentence Repetition (2 points), and (b) Verbal Fluency (1 point), 6) Abstraction (2 points), 7) Delayed recall (5 points), and 8) Orientation (6 points). The MocA scores range between 0 and 30.

Research assistants employed the MoCA test to evaluate patients’ cognitive function. The test encompasses several domains: visuospatial/executive, naming, memory, attention, language, abstraction, delayed recall, and orientation. All domains except memory are scored and summarized. The MoCA test has been identified as an especially apt tool for detecting MCI, a condition intermediate between intact cognition and dementia [36, 37]. Prior research has proposed an optimal MoCA score range of 18–24 for MCI detection, with scores ≥ 25 suggesting normal cognition [38–40]. The MoCA test was applied preoperatively to determine baseline cognitive status and repeated between 5 and 9 days after surgery. POCD was reported to present when there was a reduction of postoperative MoCA of ≥ 1 SD [41]. According to the study conducted within the Thai population, an SD of MoCA was 2.14 [42]. Consequently, a reduction in the MoCA score of ≥ 2 from baseline indicated POCD.

#### Confusion assessment method (CAM) and confusion assessment method for intensive care unit (CAM-ICU)

The optimal screening tools for POD are still a topic of debate. In our study, we utilized CAM or CAM-ICU according to our familiarity and experience with using these delirium screening tools in our institutional practice. Additionally, the CAM and CAM-ICU are recognized as high-sensitivity and high-specificity tools for screening POD [43].

The CAM or CAM-ICU tool evaluates four features: (1) sudden onset or fluctuating course, (2) inattention, (3) disorganized thinking, and (4) altered levels of consciousness. A delirium diagnosis using CAM requires the presence of features 1 and 2, along with either 3 or 4. Research assistants evaluated the patients daily from the first to the third postoperative day. The CAM tool was used for patients admitted to wards postoperatively, while CAM-ICU was used in the intensive care unit (ICU) setting. Any delirium episode occurring between postoperative days 1 and 3 was considered POD.

#### Diagnostic and statistical manual of mental disorders, fifth edition (DSM-5)

DSM-5 assesses 5 criteria: (A) Disturbance in attention, (B) Disturbance develops over a short period of time, (C) An additional disturbance in cognition, (D) The disturbances in Criteria A and C are not better explained by a pre-existing, established or evolving neurocognitive disorder and do not occur in a context of a severely reduced level of arousal such as coma., and (E) There is evidence from history, physical examination or laboratory findings that the disturbance is a direct physiological consequence of another medical condition, substance intoxication or withdrawal, or exposure to a toxin, or is due to multiple etiologies. The delirium diagnosis requires meeting all 5 criteria outlined in DSM-5.

DSM-5 is recognized as the gold standard for delirium diagnosis. A board-certified geriatrician confirmed POD diagnoses from postoperative patient chart reviews, physical examination, interviews with patients and their caregivers, and direct patient observation. POD was diagnosed when all DSM-5 criteria were met. Any delirium episode occurring between postoperative days 1 and 3 was considered POD, as 97% of delirium cases arise within 3 days postsurgery [44].

### Sample size calculation

Previous literature reported a 13% incidence of POD (p 0.13) in patients with preexisting MCI compared to a 2% incidence (p 0.02) in cognitively normal cases after anesthesia [27]. The patient database from the Department of Anesthesiology, Faculty of Medicine Siriraj Hospital, revealed a 1:4 ratio of non-MCI to MCI elderly patients during the preoperative phase. Given the 2 independent proportions and accounting for a type 1 error (α) of 0.05 and a type 2 error (β) of 0.2, the projected sample sizes for non-MCI and MCI were set at 55 and 220, respectively.

### Statistical analysis

Descriptive statistics were used to report the patients’ baseline and clinical characteristics. Data normality was assessed using the Kolmogorov–Smirnov or Shapiro–Wilk test. Categorical variables were analyzed using the chi-square or Fisher’s exact test and are reported as frequency (n) and percentage (%). Continuous data were analyzed using Student’s *t*-test for independent samples or the Mann–Whitney *U* test based on their distributions and are presented as the mean ± standard deviation or median and interquartile range. Because of missing data in postoperative MoCA scores for some subjects in both non-MCI and MCI groups, we were left with partially paired data. To address this, we followed statistical recommendations and employed a complete-case analysis approach, conducting statistical tests for POCD only on subjects with complete data (naïve paired test) [45]. All tests were 2-tailed with an α error < 5%. A probability (*P*) value < 0.05 was considered statistically significant. The analyses were executed using IBM SPSS Statistics, version 26 (IBM Corp, Armonk, NY, USA).

## Results

**Fig. 1:**
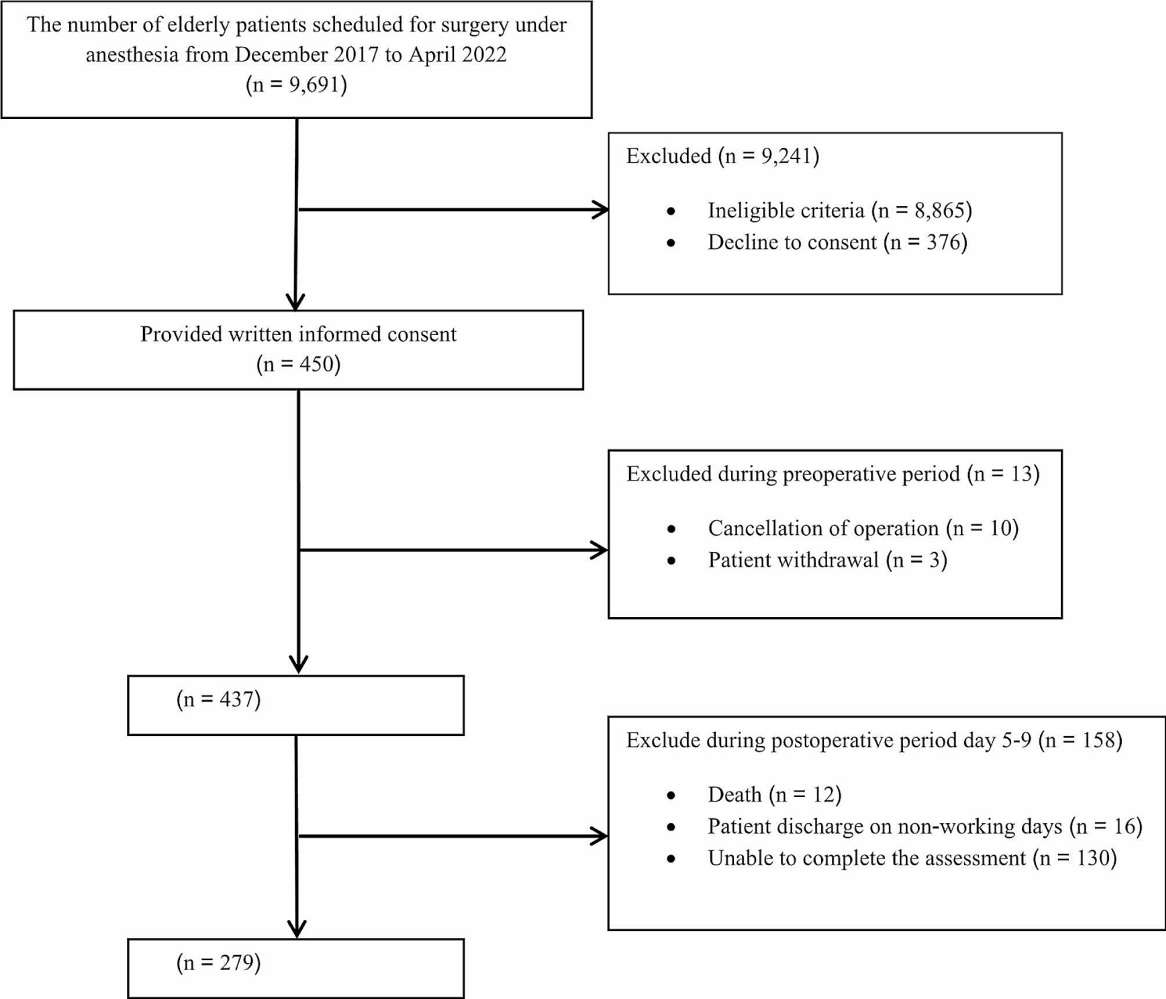
Consort flow

Between December 2017 and April 2022, 9,691 elderly individuals were scheduled for major surgery under anesthesia. A total of 8,865 ineligible patients and 376 patients who declined consent were excluded. The participants who agreed to consent (450) were further excluded due to various reasons such as cancellation of operation, patient withdrawal, postoperative death, and failure to retrieve complete assessments. A total of 279 participants proceeded to final analysis (Fig. 1). The patients were categorized into baseline preoperative non-MCI and MCI groups, comprising 56 and 223 individuals, respectively. The cohort was predominantly male and had an average age of approximately 70. The preoperative demographic profiles of the 2 groups showed many similarities such as weight, height, American Society of Anesthesiologists (ASA) physical status, comorbidities, history of alcohol consumption and smoking, benzodiazepine use, site of surgery, and timing of surgery. The non-MCI group demonstrated a significantly higher education level and greater proportions of ischemic heart disease and hyperlipidemia patients. Conversely, the MCI group contained a larger proportion of patients with congestive heart failure. Most patients were scheduled for elective surgery across various organ systems, with CVT surgery being the most frequent (Table 1). The predominant anesthesia type was general. Intraoperatively, the non-MCI group had significantly higher percentages of patients who had hypotension, required blood transfusions, or were administered midazolam than the MCI patients (Table 2). Postoperatively, the non-MCI group displayed significantly larger proportions of patients who received benzodiazepines, tricyclic antidepressants, or blood transfusions. Other postoperative profiles between the two groups were similar, including the use of postoperative opioids and antihistamines, the incidence of poorly controlled pain, anemia, blood transfusion, and other adverse events such as hypotension, cardiac arrest, arrhythmia, acute kidney injury, oxygen desaturation, etc. (Table 3).

**Table 1:** Preoperative demographic and clinical profiles of patients

Variables	Non-MCI (*n* = 56)	MCI (*n* = 223)	*P*
Sex			0.448
Male	30 (53.6%)	134 (60.1%)	
Female	26 (46.4%)	89 (39.9%)	
Age (years)	70.82 ± 7.06	71.19 ± 6.51	0.722
Age range (years)			0.625
60–69	24 (42.9%)	81 (36.3%)	
70–79	25 (44.6%)	115 (51.6%)	
≥ 80	7 (12.5%)	27 (12.1%)	
Weight (kg)	62.36 ± 10.54	63.95 ± 11.18	0.324
Height (cm)	159.03 ± 8.56	160.64 ± 7.90	0.204
Body mass index (kg/m^2^)	24.68 ± 3.88	24.78 ± 4.05	0.856
Education Level			< 0.001*
≤ 12 years	25 (44.6%)	159 (71.3%)	
> 12 years	31 (55.4%)	64 (28.7%)	
ASA physical status			0.072
ASA < 3	11 (19.6%)	73 (32.7%)	
ASA ≥ 3	45 (80.4%)	150 (67.3%)	
Comorbidities			
Neurological system			
Cerebrovascular accident	5 (8.9%)	24 (10.8%)	0.811
Depression	1 (1.8%)	4 (1.8%)	1.000
Paraplegia	0 (0.0%)	1 (0.4%)	1.000
Hemiplegia	0 (0.0%)	4 (1.8%)	0.588
Cardiovascular system			
Hypertension	42 (75.0%)	172 (77.1%)	0.859
Atrial fibrillation	9 (16.1%)	29 (13.0%)	0.516
Congestive heart failure	1 (1.8%)	28 (12.6%)	0.014*
Ischemic heart disease	33 (58.9%)	89 (39.9%)	0.015*
Valvular heart disease	13 (23.2%)	46 (20.6%)	0.713
Peripheral vascular disease	1 (1.8%)	5 (2.2%)	1.000
Endocrine system			
Hyperlipidemia	44 (78.6%)	141 (63.2%)	0.030*
Hypothyroidism	2 (3.6%)	6 (2.7%)	0.660
Hyperthyroidism	2 (3.6%)	2 (0.9%)	0.177
Type 2 diabetes mellitus	20 (35.7%)	60 (26.9%)	0.186
Respiratory system			
Asthma	0 (0.0%)	5 (2.2%)	0.587
Recent upper respiratory tract infection	1 (1.8%)	1 (0.4%)	0.359
Chronic obstructive pulmonary disease	2 (3.6%)	3 (1.3%)	0.259
Renal system			
Chronic kidney disease stage 1	8 (14.3%)	28 (12.6%)	0.659
Chronic kidney disease stage 2	29 (51.8%)	113 (50.7%)	0.879
Chronic kidney disease stage 3	9 (16.1%)	46 (20.6%)	0.572
Chronic kidney disease stage 4	6 (10.7%)	21 (9.4%)	0.799
Others			
Malignancy	11 (19.6%)	67 (30.0%)	0.180
Metastatic lesion	1 (1.8%)	13 (5.8%)	0.315
Rheumatic disease	7 (12.5%)	23 (10.3%)	0.629
Skin ulcer	0 (0.0%)	1 (0.4%)	1.000
Liver disease	1 (1.8%)	8 (3.6%)	1.000
Peptic ulcer	1 (1.8%)	1 (0.4%)	0.358
Electrolyte imbalance	2 (3.6%)	9 (4.0%)	1.000
Alcohol use	3 (5.4%)	13 (5.8%)	1.000
Smoking	2 (3.6%)	2 (0.9%)	0.181
Visual impairment	32 (57.1%)	107 (48.0%)	0.235
Auditory impairment	5 (8.9%)	10 (4.5%)	0.195
Benzodiazepine premedication			
Diazepam	0 (0.0%)	2 (0.9%)	1.000
Lorazepam	1 (1.8%)	17 (7.6%)	0.135
Site of surgery			0.096
Cardiovascular-thoracic	38 (67.9%)	122 (54.7%)	
Non-cardiovascular-thoracic	18 (32.1%)	101 (45.3%)	
- Neurological	0 (0.0%)	1 (0.4%)	
- Colorectal	2 (3.6%)	20 (9.0%)	
- Hepatobiliary	3 (5.4%)	5 (2.2%)	
- Gynecological	1 (1.8%)	8 (3.6%)	
- Urological	4 (7.1%)	21 (9.4%)	
- Orthopedic	8 (14.3%)	46 (20.6%)	
Timing of surgery			0.644
Elective	53 (94.6%)	215 (96.4%)	
Urgency	3 (5.4%)	7 (3.1%)	
Emergency	0 (0.0%)	1 (0.4%)	

ASA, American Society of Anesthesiologists; MCI, mild cognitive impairment; MoCA, Montreal Cognitive Assessment

*Significance at *P* < 0.05

**Table 2:** Characteristics of intraoperative procedures

Variables	Non-MCI (*n* = 56)	MCI (*n* = 223)	*P*
Anesthesia technique			0.206
General anesthesia	48 (85.7%)	171 (76.7%)	
Regional anesthesia	5 (8.9%)	21 (9.4%)	
Combined	3 (5.4%)	31 (13.9%)	
Hypotension	15 (26.8%)	26 (11.7%)	0.010*
Arrhythmia requiring defibrillation	1 (1.8%)	3 (1.3%)	1.000
Benzodiazepine use^a^	14 (25.0%)	9 (4.0%)	< 0.001*
Blood transfusion	15 (26.8%)	13 (5.8%)	< 0.001*
Duration of operation (min)	260 (167.5–330)	225 (137.5–330)	0.303

a. Midazolam

MCI: mild cognitive impairment; min: minutes

*Significance at *P* < 0.05

**Table 3:** Postoperative patient conditions

Variables	Non-MCI (*n* = 56)	MCI (*n* = 223)	*P*
Benzodiazepine use^a^	11 (19.6%)	20 (9.0%)	0.032*
Opioid use^b^	4 (7.1%)	9 (4.0%)	0.302
Tricyclic antidepressant use^c^	3 (5.4%)	0 (0.0%)	0.008*
Antihistamine use^d^	1 (1.8%)	3 (1.3%)	1.000
Poorly controlled pain	5 (8.9%)	10 (4.5%)	0.192
Anemia	7 (12.5%)	12 (5.4%)	0.074
Blood transfusion	7 (12.5%)	3 (1.3%)	0.001*
Hypotension	3 (5.4%)	7 (3.1%)	0.425
Cardiac arrest	1 (1.8%)	8 (3.6%)	0.692
Myocardial infarction	1 (1.8%)	2 (0.9%)	1.000
Arrhythmia	11 (19.6%)	50 (22.4%)	0.856
Coma	1 (1.8%)	8 (3.6%)	0.692
Cerebrovascular accident	0 (0.0%)	6 (2.7%)	0.602
Convulsion	0 (0.0%)	4 (1.8%)	0.587
Acute kidney injury	4 (7.1%)	28 (12.6%)	0.349
Urinary retention	1 (1.8%)	4 (1.8%)	1.000
Urinary tract infection	0 (0.0%)	10 (4.5%)	0.133
Oxygen desaturation	4 (7.1%)	18 (8.1%)	1.000
Pneumonia	0 (0.0%)	13(5.8%)	0.078
Pulmonary embolism	0 (0.0%)	2 (0.9%)	1.000
Re-operation	3 (5.4%)	10 (4.5%)	1.000
Death	0 (0.0%)	9 (4.0%)	0.212

(a) Lorazepam, clonazepam, diazepam, midazolam; (b) Tramadol, pethidine; (c) Amitriptyline, Nortriptyline; (d) First generation antihistamines: chlorpheniramine, hydroxyzine, diphenhydramine

MCI: mild cognitive impairment

*Significance at *P* < 0.05

The 2 groups had comparable postoperative ICU admission rates, durations of ICU stay, dependence on mechanical ventilators, lengths of hospital stay, and healthcare costs. Postoperative neurocognitive status, reflected in the incidences of POD and POCD, was assessed. POD diagnoses were made by research assistants using the CAM and confirmed with the DSM-5 criteria by a geriatrician. The incidence of POD in the non-MCI and MCI groups was 14.3% and 16.6%, respectively, with no significant difference observed. POCD assessments involved fewer patients in both groups (46 and 173 patients in the non-MCI and MCI groups, respectively) due to some patients not being available for postoperative MoCA scoring. Notably, the incidence of POCD in the non-MCI group was 50%, twice as high as that in the MCI group. Moreover, patients with prior POD diagnoses had a significantly higher likelihood of developing POCD than those without POD, with rates of 63.6% versus 23.7%, respectively (Table 4).

**Table 4:** Patient progress postsurgery

Variables	Non-MCI (*n* = 56)	MCI (*n* = 223)	*P*
ICU admission			0.072
Yes	31 (55.4%)	93 (41.7%)	
No	25 (44.6%)	130 (58.3%)	
ICU stay^a^ (days)	2 (2–3)	3 (2–4)	0.407
Postoperative ventilator use			0.052
Yes	31 (55.4%)	91 (40.8%)	
No	25 (44.6%)	132 (59.2%)	
Days of ventilator^b^	1 (1-1.5)	1 (1–2)	0.141
Length of hospital stay	8 (6-11.5)	8 (6–12)	0.766
Healthcare cost^c^ (Thai Baht)	223,605 (171,454 − 336,247.5)	216,326 (142,104–316,712.3)	0.905
Neurocognitive status			
POD	8 (14.3%)	37 (16.6%)	0.839
POCD^d^	23 (50%)	42 (24.3%)	0.001*
- Preoperative MoCA	26.54 ± 1.44	20.89 ± 1.89	< 0.001*
- Postoperative MoCA^d^	23.85 ± 4.92	21.25 ± 3.64	0.001*
Patients with preceding POD^e^	**No (n = 186)**	**Yes (n = 33)**	
Subsequent POCD^e^	44 (23.7%)	21 (63.6%)	< 0.001*

(a) Total *n* = 124: non-MCI = 31, MCI = 93. (b) Total *n* = 122: non-MCI = 31, MCI = 91. (c) Total *n* = 277: non-MCI = 56, MCI = 221. (d) Total *n* = 219: non-MCI = 46, MCI = 173. (e) Patients who had complete data on pre- and postoperative MoCA scores, Total *n* = 219: no preceding POD = 186, with preceding POD = 33.

ICU, intensive care unit; MCI, mild cognitive impairment; MoCA, Montreal Cognitive Assessment; POCD, postoperative cognitive dysfunction; POD, postoperative delirium

*Significance at *P* < 0.05

Upon subsequent stratification and analysis of MoCA domains, significant postoperative declines were identified in the non-MCI group across the visuospatial, naming, attention, language, abstraction, and orientation domains. For the MCI group, significant decreases were observed solely in the visuospatial, attention, and orientation domains. Interestingly, while the non-MCI group**’**s pre- and postoperative scores for the delayed recall domain remained consistent, the MCI group saw a significant increase. The overall postoperative MoCA score of the non-MCI group significantly decreased from its preoperative baseline, whereas the MCI group**’**s pre- and postoperative scores remained stable (Table 5).

**Table 5:** Pre- and postoperative MoCA domain score comparisons between non-MCI and MCI patients

Domain	Non-MCI			MCI		
	Preop *n* = 56	Postop *n* = 46	*P*	Preop *n* = 223	Postop *n* = 173	*P*
Visuospatial	4.20 ± 0.83	3.59 ± 1.38	0.001*	3.18 ± 1.12	2.89 ± 1.33	0.001*
Naming	2.93 ± 0.33	2.83 ± 0.57	0.024*	2.87 ± 0.37	2.88 ± 0.37	0.671
Attention	5.74 ± 0.49	5.22 ± 1.23	0.006*	5.08 ± 0.97	4.84 ± 1.10	0.008*
Language	2.46 ± 0.66	1.93 ± 1.02	< 0.001*	1.13 ± 0.89	1.22 ± 0.98	0.244
Abstraction	1.54 ± 0.66	1.24 ± 0.85	0.021*	0.53 ± 0.70	0.58 ± 0.72	0.190
Delayed recall	3.65 ± 1.29	3.70 ± 1.35	0.854	1.96 ± 1.44	3.13 ± 1.59	< 0.001*
Orientation	5.91 ± 0.29	5.20 ± 1.03	< 0.001*	5.72 ± 0.57	5.31 ± 0.91	< 0.001*
Total MoCA	26.54 ± 1.44	23.85 ± 4.92	0.001*	20.89 ± 1.89	21.25 ± 3.64	0.243

MCI, mild cognitive impairment; MoCA, Montreal Cognitive Assessment; Postop, postoperative; Preop, preoperative

*Significance at *P* < 0.05

## Discussion

With the global increase in the aging population, there is a concomitant rise in the demand for surgery and anesthesia. The elderly population is viewed as vulnerable due to their susceptibility to postoperative complications, notably various cognitive disorders.

Delirium is the most common cognitive complication postsurgery. Recent findings indicate that its etiology involves both patient and surgical factors. Baseline factors predisposing to POD are a lower educational level, alcohol abuse, an American Society of Anesthesiologists physical status ≥ 3, prior stroke incidents, congestive heart failure, and myocardial infarction. Hyperlipidemia, diabetes mellitus, and preexisting dementia also contribute to POD [43–50]. Regarding operative parameters, delirium onset is influenced by procedure duration, length of hospitalization, intraoperative hypotension or blood transfusion, and specific medications (benzodiazepine, tricyclic antidepressants, and opioids) [47, 51–55]. The risk factors for POCD largely parallel those of delirium, particularly lower educational level, ischemic heart disease, intraoperative hypotension or blood transfusion, and the administration of benzodiazepines and tricyclic antidepressants [47, 56–58]. Furthermore, a lower preoperative cognitive score has been linked to both the emergence of POD and subsequent POCD development [18].

Our research employed the MoCA test to detect preexisting MCI. This instrument has been demonstrated to be superior to other neurocognitive tests, such as the MMSE, in MCI screening [32, 36]. The MoCA test has also demonstrated high sensitivity and specificity for detecting POCD [36]. Delirium was assessed using the CAM or the CAM-ICU, with a seasoned geriatrician confirming the results based on DSM-5 criteria. The significantly different perioperative demographics between the non-MCI and MCI patients in our study align with previously reported risk factors for POD.

In our cohort, the overall incidence of POD was 16.13% (45 out of 279 patients), with similar incidence rates in the preexisting non-MCI and MCI groups. This incidence aligns with global figures [59] and previous studies from our institution [60]. Interestingly, half of the non-MCI group developed POCD, compared to approximately 25% of the MCI group. This observation may be attributed to two factors: (1) the absence of a floor effect in MoCA detection, and (2) a greater likelihood of a more pronounced reduction in postoperative MoCA scores among non-MCI patients who commenced with higher preoperative MoCA scores. Nevertheless, our findings align with previous research, supporting the notion that individuals with normal cognition face an elevated risk of early POCD and thus requiring close attention [30]. Consistent with earlier studies, our data indicate that having a POD episode increases the risk of developing POCD [18]. A domain-specific MoCA analysis revealed a significant decline in most domains from baseline for the non-MCI group, highlighting their predisposition to POCD. In contrast, the MCI group exhibited a surprising postoperative boost in the delayed recall domain. A potential reason could be the “practice effect” of the MoCA test, given prior findings suggesting its vulnerability to this effect even with 12-month gaps between assessments [61]. However, why this effect is observed in only a single domain remains unclear.

Given the high incidence—up to 50%—of POD and POCD following cardiovascular surgery [62–64], we found it compelling to investigate whether CVT surgery plays a role in developing POCD. A decrease in postoperative MoCA scores was noted in the visuospatial, attention, and orientation domains among CVT patients, whereas only the orientation domain showed a decline in the non-CVT group. Although there was a noticeable reduction in the overall postoperative MoCA score in the CVT group compared to the baseline, it was not statistically significant (*P* = 0.081; Supplementary Table 1). This suggests that preoperative non-MCI status and CVT surgery might influence the onset of POCD. This finding was corroborated when examining both MCI and CVT surgery together. Non-MCI patients who underwent CVT surgery displayed a significant reduction in total postoperative MoCA score, with lower postoperative scores in the visuospatial, attention, language, abstraction, and orientation domains, suggesting a link to POCD (Supplementary Table 2).

As previously mentioned, several perioperative parameters—not just MCI—might have affected our study**’**s incidence of POD and POCD. Consequently, we performed an association analysis to ensure the appropriateness of our findings. Patients were categorized into non-POD and POD or non-POCD and POCD groups. Subsequently, the relationships between MCI or CVT surgery and either POD or POCD were analyzed. MCI did not correlate with POD. However, MCI was more likely associated with the absence of POCD. Interestingly, CVT surgery correlated with the development of both POD and POCD (Supplementary Table 3).

In the context of MoCA domains, given the lack of prior investigations exploring variations in postoperative scores across MoCA domains indicative of MCI, we deemed it pertinent to contribute valuable insights to the field by undertaking analyses in this regard. A previous study assessed the diagnostic accuracy of MoCA subsections in Parkinson’s disease patients, both with and without cognitive impairment, by comparing them with a comprehensive neuropsychological battery. The findings indicated that the executive performance component of MoCA, encompassing visuospatial/executive and abstraction domains, exhibited 90% sensitivity in detecting cognitive impairment. However, the sensitivity in assessing language or attention impairment was limited [65]. MoCA, characterized by a collection of concise neuropsychological tests, offers advantages in terms of time efficiency and practicality compared to lengthier neuropsychological assessments. Nevertheless, the crucial consideration of domain-specific screening becomes evident, given the divergent diagnostic performance of MoCA domains. Our study’s results pertaining to visuospatial and abstraction subsections revealed a more pronounced reduction in postoperative scores from baseline in non-MCI and CVT patients, suggesting a predisposition to POCD in these groups. Consequently, postoperative follow-up becomes imperative to monitor the improvement of MoCA scores, especially in the context of domain-specific impairments.

Another study reported the subtype of MCI called executive MCI, where patients developed impairment of executive function without a memory deficit. This condition poses a risk for the development of Alzheimer’s disease [66]. Considering the fact that the executive domain is the most sensitive, further follow-up and evaluation are important to track the progression or recovery of the patients. Furthermore, it could be necessary to conduct additional research to investigate the relationship between anesthesia, the type of surgery, and alterations in these MoCA domains.

Our study does have limitations. First, MCI screening was not performed per the International Working Group for MCI or DSM-5 guidelines. Instead, we utilized the MoCA test, a more suitable tool for MCI screening than others, such as the MMSE [32, 57, 66]. Recent research also supported our approach of classifying MCI as a MoCA score between 18 and 24: the study demonstrated sensitivity and specificity rates exceeding 80% [38]. Another limitation was that approximately 20% of data on the postoperative MoCA scores of our participants were missing. This absence may have skewed our interpretation of the POCD incidence. A further concern was the MoCA test**’**s **“**practice effect**”**, as previously mentioned. We administered the test 1 day before surgery and then postoperatively between days 5–9. Future research would benefit from minimizing loss to follow-up rates and incorporating more extended cognitive evaluations using the MoCA test.

Despite these limitations, the strengths of our study are apparent in our use of appropriate assessment tools—CAM/CAM-ICU, MoCA, and DSM-5—and the comprehensive data collection on patients**’** perioperative characteristics. Notably, this is the first study to report changes in postoperative MoCA scores by domain. Furthermore, the information about the high incidence of POCD, even in preoperatively cognitively intact elderly individuals, had contributed to an increased awareness of the importance of close neurocognitive monitoring and follow-up. Our results underscore the critical nature of postoperative neurocognitive evaluations in patients, regardless of their MCI status, especially those who have undergone CVT surgery.

## Conclusions

POD and POCD are common postoperative complications. Our findings demonstrate comparable incidences of POD among patients with baseline MCI and cognitively intact individuals scheduled for various types and urgency levels of surgery. However, early postoperative POCD incidence was higher among non-MCI patients. Our results highlight the need for preoperative cognitive assessments and postoperative cognitive follow-ups for all elderly individuals scheduled for surgery.

### Electronic supplementary material

Below is the link to the electronic supplementary material.

**Supplementary Material 1: Supplementary Table 1** Pre- and postoperative MoCA domain score comparisons between non-CVT and CVT surgery patients. **Supplementary Table 2** Comparative analysis of pre- and postoperative MoCA domain scores between MCI and CVT patients. **Supplementary Table 3** Correlation of MCI and CVT surgery with incidences of POD and POCD

## Data Availability

The original findings of this research are incorporated in the article/Supplementary material. Additional enquiries can be directed to the corresponding author.

## Abbreviations

ASA: American Society of Anesthesiologists physical status
CAM: Confusion Assessment Method
CAM-ICU: Confusion Assessment Method for the Intensive Care Unit
CVT: Cardiovascular-thoracic
DSM-5: Diagnostic and Statistical Manual of Mental Disorders, Fifth Edition
ICU: Intensive care unit
MCI: Mild cognitive impairment
MMSE: Mini-Mental State Examination
MoCA: Montreal Cognitive Assessment
POD: Postoperative delirium
POCD: Postoperative cognitive dysfunction
