# Supplementary material for: Early postoperative neurocognitive complications in elderly patients: comparing those with and without preexisting mild cognitive impairment– a prospective study
Source: BMC Geriatr. 2024 Jan 22;24:84. doi: 10.1186/s12877-024-04663-5 (PMC10804619; doi:10.1186/s12877-024-04663-5)
Supplement: Supplementary file 1 — Supplementary Material 1: Supplementary Table 1 Pre- and postoperative MoCA domain score comparisons between non-CVT and CVT surgery patients. Supplementary Table 2 Comparative analysis of pre- and postoperative MoCA domain scores between MCI and CVT patients. Supplementary Table 3 Correlation of MCI and CVT surgery with incidences of POD and POCD [file 12877_2024_4663_MOESM1_ESM.docx]

**Supplementary Table 1** Pre- and postoperative MoCA domain score comparisons between non-CVT and CVT surgery patients

| **Domain** | **Non-CVT surgery** | | | **CVT surgery** | | |
| --- | --- | --- | --- | --- | --- | --- |
|  | **Preop**  **n = 119** | **Postop**  **n = 93** | ***P*** | **Preop**  **n = 160** | **Postop**  **n = 126** | ***P*** |
| Visuospatial | 3.47±1.19 | 3.28±1.26 | 0.264 | 3.36±1.11 | 2.86±1.42 | 0.001* |
| Naming | 2.91±0.29 | 2.92±0.30 | 0.678 | 2.86±0.45 | 2.83±0.49 | 0.682 |
| Attention | 5.09±0.97 | 5.01±1.05 | 0.562 | 5.18±0.96 | 4.85±1.20 | 0.014* |
| Language | 1.38±1.01 | 1.44±1.08 | 0.666 | 1.47±1.00 | 1.32±0.99 | 0.202 |
| Abstraction | 0.71±0.77 | 0.77±0.78 | 0.574 | 0.82±0.82 | 0.68±0.81 | 0.161 |
| Delayed recall | 2.24±1.55 | 3.18±1.66 | <0.001* | 2.34±1.59 | 3.30±1.48 | <0.001* |
| Orientation | 5.76±0.52 | 5.51±0.78 | 0.008* | 5.73±0.54 | 5.12±1.01 | <0.001* |
| Total MoCA | 21.90±2.59 | 22.42±3.82 | 0.262 | 22.12±3.11 | 21.33±4.21 | 0.081 |

CVT: Cardiovascular-thoracic surgery; MoCA: Montreal Cognitive Assessment; Postop: postoperative; Preop: preoperative

*Significance at *P* < 0.05.

**Supplementary Table 2** Comparative analysis of pre- and postoperative MoCA domain scores between MCI and CVT patients

| **Domain** | **Non-MCI** | | | | | | **MCI** | | | | | |
| --- | --- | --- | --- | --- | --- | --- | --- | --- | --- | --- | --- | --- |
|  | **Non-CVT** | | | **CVT** | | | **Non-CVT** | | | **CVT** | | |
|  | **Preop**  **n = 18** | **Postop**  **n = 14** | ***P*** | **Preop**  **n = 38** | **Postop**  **n = 32** | ***P*** | **Preop**  **n = 101** | **Postop**  **n = 79** | ***P*** | **Preop**  **n = 122** | **Postop**  **n = 94** | ***P*** |
| Visuospatial | 4.39±0.78 | 4.29±0.73 | 0.702 | 4.13±0.81 | 3.28±1.49 | 0.006* | 3.31±1.18 | 3.10±1.26 | 0.265 | 3.11±1.08 | 2.71±1.37 | 0.021* |
| Naming | 3.00±0.00 | 3.00±0.00 | N/A | 2.92±0.36 | 2.75±0.67 | 0.203 | 2.89±0.31 | 2.91±0.33 | 0.675 | 2.84±0.47 | 2.86±0.40 | 0.667 |
| Attention | 5.61±0.61 | 5.57±0.51 | 0.843 | 5.74±0.50 | 5.06±1.41 | 0.014* | 5.00±1.00 | 4.91±1.09 | 0.575 | 5.00±1.00 | 4.78±1.12 | 0.130 |
| Language | 2.61±0.61 | 2.50±0.94 | 0.705 | 2.47±0.65 | 1.69±0.97 | <0.001* | 1.16±0.90 | 1.25±0.99 | 0.510 | 1.16±0.88 | 1.19±0.97 | 0.780 |
| Abstraction | 1.44±0.78 | 1.64±0.50 | 0.390 | 1.58±0.64 | 1.06±0.91 | 0.010* | 0.58±0.70 | 0.62±0.70 | 0.732 | 0.58±0.73 | 0.55±0.73 | 0.773 |
| Delayed recall | 2.89±1.45 | 3.93±1.14 | 0.031* | 3.79±1.14 | 3.59±1.43 | 0.535 | 2.13±1.55 | 3.05±1.71 | <0.001* | 1.89±1.43 | 3.20±1.49 | <0.001* |
| Orientation | 6.00±0.00 | 5.71±0.61 | 0.104 | 5.89±0.31 | 4.97±1.09 | <0.001* | 5.73±0.55 | 5.46±0.80 | 0.009* | 5.67±0.58 | 5.17±0.98 | <0.001* |
| Total MoCA | 26.11±1.32 | 26.71±1.38 | 0.223 | 26.74±1.47 | 22.59±5.39 | <0.001* | 21.15±1.97 | 21.66±3.61 | 0.260 | 20.68±1.81 | 20.90±3.66 | 0.587 |

CVT: Cardiovascular-thoracic surgery; MCI: Mild cognitive impairment; MoCA: Montreal Cognitive Assessment; N/A: cannot be computed due to equal means and standard deviations between groups; Postop: postoperative; Preop: preoperative

*Significance at *P* < 0.05.

**Supplementary Table 3** Correlation of MCI and CVT surgery with incidences of POD and POCD

| **Variables** | **No POD**  **n = 234** | **POD**  **n = 45** | ***P*** | **No-POCD**  **n = 154** | **POCD**  **n = 65** | ***P*** |
| --- | --- | --- | --- | --- | --- | --- |
| MCI | 186 (79.5%) | 37 (82.2%) | 0.675 | 131 (85.1%) | 42 (64.6%) | 0.001* |
| CVT surgery | 123 (52.6%) | 37 (82.2%) | <0.001* | 79 (51.3%) | 47 (72.3%) | 0.004* |

CVT, cardiovascular-thoracic surgery; MCI, mild cognitive impairment; MoCA, Montreal Cognitive Assessment; POCD, postoperative cognitive dysfunction; POD, postoperative delirium

*Significance at *P* < 0.05.
